# Polarization of Type 1 Macrophages Is Associated with the Severity of Viral Encephalitis Caused by Japanese Encephalitis Virus and Dengue Virus

**DOI:** 10.3390/cells10113181

**Published:** 2021-11-15

**Authors:** Ming-Kai Jhan, Chia-Ling Chen, Ting-Jing Shen, Po-Chun Tseng, Yung-Ting Wang, Rahmat Dani Satria, Chia-Yi Yu, Chiou-Feng Lin

**Affiliations:** 1 Graduate Institute of Medical Sciences, College of Medicine, Taipei Medical University, Taipei 110, Taiwan; williamjhan2730@gmail.com (M.-K.J.); tjshen410@gmail.com (T.-J.S.); 2 Department of Microbiology and Immunology, School of Medicine, College of Medicine, Taipei Medical University, Taipei 110, Taiwan; pctseng@tmu.edu.tw (P.-C.T.); olivia0717@tmu.edu.tw (Y.-T.W.); dr.dani.satria@gmail.com (R.D.S.); 3 School of Respiratory Therapy, College of Medicine, Taipei Medical University, Taipei 110, Taiwan; chialing811@gmail.com; 4 Core Laboratory of Immune Monitoring, Office of Research & Development, Taipei Medical University, Taipei 110, Taiwan; 5 International Ph.D. Program in Medicine, College of Medicine, Taipei Medical University, Taipei 110, Taiwan; 6 Laboratory Medicine, Department of Clinical Pathology, Faculty of Medicine, Public Health and Nursing, Universitas Gadjah Mada, Yogyakarta 55281, Indonesia; 7 Clinical Laboratory Installation, Dr. Sardjito Central General Hospital, Yogyakarta 55281, Indonesia; 8 National Institute of Infectious Diseases and Vaccinology, National Health Research Institutes, Miaoli 350, Taiwan; epitope@nhri.org.tw

**Keywords:** flavivirus, dengue virus, encephalitis, macrophage, polarization

## Abstract

Infection with flaviviruses causes mild to severe diseases, including viral hemorrhagic fever, vascular shock syndrome, and viral encephalitis. Several animal models explore the pathogenesis of viral encephalitis, as shown by neuron destruction due to neurotoxicity after viral infection. While neuronal cells are injuries caused by inflammatory cytokine production following microglial/macrophage activation, the blockade of inflammatory cytokines can reduce neurotoxicity to improve the survival rate. This study investigated the involvement of macrophage phenotypes in facilitating CNS inflammation and neurotoxicity during flavivirus infection, including the Japanese encephalitis virus, dengue virus (DENV), and Zika virus. Mice infected with different flaviviruses presented encephalitis-like symptoms, including limbic seizure and paralysis. Histology indicated that brain lesions were identified in the hippocampus and surrounded by mononuclear cells. In those regions, both the infiltrated macrophages and resident microglia were significantly increased. RNA-seq analysis showed the gene profile shifting toward type 1 macrophage (M1) polarization, while M1 markers validated this phenomenon. Pharmacologically blocking C-C chemokine receptor 2 and tumor necrosis factor-α partly retarded DENV-induced M1 polarization. In summary, flavivirus infection, such as JEV and DENV, promoted type 1 macrophage polarization in the brain associated with encephalitic severity.

## 1. Introduction

Flavivirus, a genus of arthropod-borne virus that belongs to the *Flaviviridae* family, contains almost 73 viruses. Flaviviruses, such as dengue virus (DENV), Japanese encephalitis virus (JEV), and Zika virus (ZIKV), can be transmitted through mosquitoes and cause one million infections annually [1]. The flavivirus viral genome is a single-stranded, positive-sense RNA genome, approximately 11 kb in length that encodes three structural proteins (capsid (C), premembrane (prM), and envelope (E) proteins) that participate in viral attachment and maturation processes. In contrast, seven nonstructural proteins (NS1, NS2AM, NS2B, NS3, NS4A, NS4B, and NS5) are involved in viral replication and host immune response inhibition [2,3,4,5].

Flavivirus infection in patients causes symptoms including fever, headache, and vomiting [6]. However, some flaviviruses mainly cause encephalitis symptoms, such as JEV, West Nile virus (WNV), and tick-borne encephalitis virus (TBEV) [7,8,9]. However, in the past decade, WHO has included DENV infection in the central nervous system (CNS) into the diagnostic criteria of severe dengue [10], suggesting that DENV infection can cause encephalitis-like symptoms. The mechanism of how DENV causes neuropathogenesis remains unclear. Several DENV-infected rodent models show viral protein expression in the brain accompanied by immune activation [11,12,13,14]. Furthermore, brain-resident microglia have also been reported to be infected by DENV in vitro and in vivo [12,15,16]. Pharmacological blockade of microglia-derived tumor necrosis factor (TNF)-α expression further shows cytoprotection from DENV-induced neurotoxicity [17]. These studies suggest that microglial activation may play a pathogenic role in CNS inflammation during viral infection. In contrast, microglial depletion does not reduce disease symptoms or mortality, but instead promotes disease progression and the onset of mortality, indicating that microglial activation is essential for the CNS immune defense against viral infection [12,18].

Microglial activation may be multifunctional due to its regulation of neuronal survival, maintenance of immune homeostasis, and injury tissue repair in the CNS [19,20,21]. However, the microglial function can be diverse depending on the stimulation. For instance, classical activation, also known as the type 1 microglia/macrophage (M1) phenotype, is stimulated by proinflammatory mediators such as TNF-α and interleukin (IL)-6 [22,23]. Polarization of M1 microglia/macrophages may participate in cytokine secretion and antigen presentation for immune defense and cause unfavorable effects by triggering neurotoxicity and neurodegeneration [22,23,24]. Alternative activation, known as the M2 phenotype, can provide immunomodulation to repair damaged neuronal tissue by controlling inflammation [25]. Flavivirus infections are reported to cause blood-brain barrier (BBB) destruction [26,27,28,29,30]. Previous reports indicate kinetic changes in peripheral macrophage infiltration and inflammatory factor secretion in the brain after DENV infection [31]. According to the previous experimental encephalitis-like models of DENV infection in the immunocompetent mice [12,15,16], similar and conditional procedures of the infectious route have been performed to investigate immune activation and microglia/macrophage polarization during flavivirus-induced encephalitis-like disease.

## 2. Materials and Methods

### 2.1. Cells, Virus Strains, and Reagents

Baby hamster kidney (BHK)-21 cells (ATCC^®^ CCL-10™) were cultured and maintained in Dulbecco’s Modified Eagle’s Medium (DMEM; Invitrogen Life Technologies, Waltham, MA, USA) containing 10% fetal bovine serum (FBS) (Sigma-Aldrich, St. Louis, MO, USA) at 37 °C with 5% CO_2_. In addition, *Aedes albopictus* C6/36 cells (ATCC^®^ CRL-1660™) were grown in Minimum Essential Media (MEM; Invitrogen Life Technologies) supplemented with 10% FBS. The C6/36 cells were cultured in a T75 flask and prepared for service as flavivirus amplification hosts. DENV2 PL046, a Taiwanese human isolate strain, was obtained from the Centers for Disease Control in Taiwan. JEV RP-9, a Taiwanese human isolated strain, was obtained from Dr. Y. L. Lin (Institute of Biomedical Sciences, Academia Sinica, Taipei, Taiwan). ZIKV, a Puerto Rican human isolated strain, was obtained from Dr. C. Y. Yu. (National Institute of Infectious Diseases and Vaccinology, National Health Research Institutes, Tainan, Taiwan). All flaviviruses were amplified in C6/36 cells, and viral titers were quantified by plaque assay using BHK-21 cells.

### 2.2. Antibody and Reagents

The antibodies and reagents used in this study are listed below: neutralizing antibodies against mouse TNF-α (clone MP6-XT22; BioLegend, San Diego, CA, USA); neutralizing antibodies against mouse C-C chemokine receptor type 2 (CCR2) (Tocris, Bristol, UK); antibodies against DENV nonstructural protein 1 (NS1, Catalog #GTX124280) and nonstructural protein 3 (NS3, Catalog #GTX124252); antibodies against JEV NS1 (Catalog #GTX124280) and NS3 (Catalog #GTX1242867); antibodies against ZIKV NS1 (Catalog #GTX133323) and NS3 (Catalog #GTX133309); antibodies against NeuN (Catalog #GTX132974) and Iba1 (Catalog #GTX101495) (GeneTex, San Antonio, TX, USA); antibodies against caspase-1 (Catalog #SC514) (Santa Cruz, Dallas, TX, USA); antibodies against active caspase-3 (Catalog #9661S), horseradish peroxidase (HRP)-conjugated goat anti-rabbit IgG (Catalog #SC7074S), and HRP-conjugated horse anti-mouse IgG (Catalog #SC7076S) (Cell Signaling Technology, Danvers, MA, USA); antibodies against mannose receptor (ab64693) (Abcam, Cambridge, UK); antibodies against β-actin (Catalog #A5441) (Sigma–Aldrich, St. Louis, MO, USA); antibodies against CD11b (Catalog #69011282), CD45 (Catalog #56045182), CD80 (Catalog #63080182), CD86 (Catalog #67086282), CD163 (Catalog #17163162), CD206 (Catalog #25206182); Alexa Fluor 488-conjugated goat anti-mouse (Catalog #A11029), Alexa Fluor 488-conjugated goat anti-rabbit (Catalog #A11008), and Alexa Fluor 594-conjugated goat anti-rabbit (Catalog #A11037) (Invitrogen, Thermo Fisher Scientific, Waltham, MA, USA).

### 2.3. Animals and Flavivirus Infection In Vivo

According to guidelines established by the Ministry of Science and Technology, Taiwan, protocols were approved by the Institutional Animal Care and User Committee of National Defense Medical Center (IACUC number: 21055). Flavivirus infectious models were modified according to our previous study [12,32]. Seven-day-old ICR suckling mice were obtained from BioLASCO Taiwan Co., Ltd. (Taipei, Taiwan) and were inoculated intracerebrally with 2.5 × 10^5^ plaque-forming units (PFU) and intraperitoneally with 7.5 × 10^5^ PFU of DENV2 (PL046) or ZIKV (PRVABC-59). JEV was followed intracerebrally with 2.5 × 10^3^ PFU and intraperitoneally with 7.5 × 10^3^ PFU of JEV (RP-9). CCR2 inhibition was based on the DENV infection model and combined with or without BMS (10 mg/kg) treatment. Anti-TNFα treatment models were based on the DENV infection model and combined with or without Anti-mTNFα (250 μg/kg) treatment. All the flavivirus-infected mice were monitored, and disease progression was scored according to our previous studies [12,32]. Mice were sacrificed, and tissue was harvested when the disease score presented paralysis symptoms.

### 2.4. Plaque Assay

Mouse brains were harvested when the indicated score was present and homogenized with ice-cold phosphate-buffered saline (PBS) for supernatant collection. BHK-21 cells were resuspended at a concentration of 1 × 10^5^ cells/well in DMEM and seeded into a 12-well culture plate for further plaque assay tests. Briefly, the supernatant was serially diluted in DMEM containing 2% FBS and further incubated with monolayer BHK-21 cells at 37 °C and 5% CO2 for 2 h. After 2 h, the supernatant was discarded and filled with 0.5% methylcellulose solution (DMEM containing 4% FBS) for 5 to 7 days. Methylcellulose solutions were removed by washing, and cell pellets were stained with crystal violet for 12 to 24 h. After being discarded with crystal violet, viral plaque formations were visualized under a microscope.

### 2.5. Immunofluorescence Stain

Whole mouse brains were perfused with an ice-cold PBS and fixed in 4% neutral-buffered formalin. A fixed brain was embedded in an optimal cutting temperature compound (OCT), and cryosection (20 μm) was performed by cryostat microtome. Immunofluorescence staining was performed according to the following procedure: frozen tissue slices were removed from −20 °C and placed into room temperature for 10–20 min. Slices were further rehydrated and washed by PBS for 10 min. Tissue non-specific binding was performed by adding the blocking buffer for 1 h (PBS containing 5% bovine serum albumin). After 1 h, the tissue slices were washed twice and stained with primary antibodies against active caspase-3, the neuron marker NeuN, and the activated macrophage marker Iba-1, overnight in 4 °C. The primary antibody was diluted in antibody diluent (Dako S3022), and antibody concentration was determined according to product sheet suggestion. After one night, slices were washed twice and stained with secondary antibody for 2 h. DAPI was used for nuclear staining for 30 min. Fluorescent tissue sections were visualized under a fluorescence microscope (Ax10, Zeiss, Oberkochen, Germany).

### 2.6. Nissl Stain and Histological Stain

For histological analysis, tissue slices were performed with Nissl or hematoxylin and eosin (H&E) staining. Neuron density changes were detected by the Nissl bodies lost in the neuronal cells. Briefly, frozen tissue slices were removed and placed at room temperature for 10–20 min. After OCT melting, the slices were rinsed in ddH20 to wash out OCT and rehydrate the slices. The rehydrated slices were immediately immersed in a Nissl staining buffer (1% glacial acetic acid solution containing 1% cresyl violet) for 5 min. Slices were dehydrated with increased concentration of EtOH (30%, 50%, 70%, and 95%) for 5 min each, after being rinsed with ddH2O and mounted with mounting medium (Lecia). For H&E staining, the rehydrated tissue slices were stained with 0.1% hematoxylin for 10 min and rinsed with ddH2O. Slices were further immersed in 0.5% eosin buffer for 10 min. After rinsed and dehydrated with EtOH and mounted with a mounting medium, the stained slices were visualized under a microscope to measure neuron destruction and histological change.

### 2.7. Flow Cytometry

Mouse brains were harvested when the indicated score was present and homogenized with ice-cold Hank’s Balanced Salt Solution (HBSS, Invitrogen Life Technologies) containing 10% FBS. The cell solution was centrifuged and resuspended in a digestion buffer (Accutase, Invitrogen Life Technologies) for cell detachment and dissociation. The cell solution was centrifuged and washed with ice-cold HBSS. The solution was further centrifuged and resuspended in a 25% density gradient medium (Percoll) and centrifuged again. After centrifugation, the supernatant containing the myelin coat was discarded. Cell pellets were washed and further stained with the leukocyte marker CD45, monocyte marker CD11b, macrophage marker F4/80, M1-related markers CD80 and CD86, and M2-related markers CD163 and CD209. Staining cells were analyzed by flow cytometry (Attune Nxt, Thermo Fisher Scientific) for immune profiling.

### 2.8. Multiplex Assay

Mouse brains were harvested when the indicated score was present and homogenized with 400 μL ice-cold PBS and centrifuged at 12,000 rpm for one half-hour. According to the manufacturer’s instructions, brain supernatants were collected and quantified to the 1 mg/mL concentration (accessed on 29 October 2021. https://www.merckmillipore.com/TW/zh/product/MILLIPLEX-MAP-Mouse-TH17-Magnetic-Bead-Panel-Immunology-Multiplex-Assay,MM_NF-MTH17MAG-47K#documentation). The procedure of this assay was started by adding a 200 μL wash buffer into 96 wells and shaking for 10 min at room temperature. After removing the wash buffer, 25 μL of control/standard buffer was added into appropriate wells, and 25 μL of assay buffer was added into the sample/background wells. Furthermore, 25 μL of an appropriate matrix solution was added into background, standards, and control wells. Next, 25 μL of quantified brain supernatant and 25 μL antibody-beads were added into wells for overnight incubation in 4 °C. The solution was removed from wells and washed after the overnight incubation. Next, a 25 μL of detection antibody was added into each well for 1 h at room temperature. The 25 μL of the streptavidin-phycoerythrin solution was immediately added into each well for another 30 min of incubation. Finally, 150 μL of sheath fluid was added to each well after removing and gently washing the well contents. The protein expression level was detected and analyzed by Luminex^®^ 200TM, HTS, FLEXMAP 3DTM, or MAGPIX^®^ with xPONENT software (Millipore, Burlington, MA, USA).

### 2.9. Western Blotting

Mouse brains were harvested when the indicated score was present and homogenized with ice-cold PBS. Cell pellets were collected, and the protein concentration was quantified. Equal concentrations of proteins were separated by using SDS polyacrylamide gel electrophoresis. After 2 h, the protein was immediately transferred to a polyvinylidene difluoride membrane (PVDF, Millipore, Burlington, MA, USA). After transfer, the PVDF membrane was blocked with 5% nonfat milk for 1 h and washed twice with Tris-based saline containing 0.05% Tween 20 (TBST). The PVDF membrane was stained with the indicated antibodies (NS1, NS3, active caspase-3, caspase-1, and β-actin). The protein signal was detected with secondary antibodies and expressed by an ECL Western blot detection kit (Pierce Chemical, Rockford, IL, USA).

### 2.10. Gene Sequencing

Mouse brains were harvested when the indicated score was present and homogenized with ice-cold PBS. Cell pellets were collected and dissolved in TRIzol reagent (Gibco-BRL, Grand Island, NY, USA). Brain samples were sent to Biotools (New Taipei City, Taiwan) for further RNA isolation and mRNA sequencing (RNA-seq) analysis. RNA quality and concentration were brief. Further, sequencing libraries were performed by using the KAPA mRNA HyperPrep Kit (Roche, Basel, Switzerland). mRNA was captured by magnetic oligo-dT beads and fragmented by and co-incubated with magnesium at high temperatures. A random hexamer primer was used to form the 1st strand cDNA. A combination of second-strand synthesis and A-tailing transformed cDNA:RNA to double-stranded cDNA (dscDNA). dUTP and dAMP were bound to the second cDNA strand and dscDNA 3’end. Next, dsDNA adapters with 3’dTMP overhangs were combined with library insert fragments and formed the library fragments. The KAPA Pure Beads system (Roche, Basel, Switzerland) was used to extract the library fragments, and KAPA HiFi HotStart ReadyMix (Roche, Basel, Switzerland) was used to amplify the sequences. KAPA Pure Beads system and Qsep 100 DNA/RNA Analyzer (BiOptic Inc., New Taipei City, Taiwan) were further performed to test the library quality.

### 2.11. RNA Data Analysis

For RNA data analysis, the sequencing depth was 20 million reads. A high-throughput sequencing (Illumina NovaSeq 6000 platform) was used to transform the original data to raw sequenced reads file (pairs reads = pe150), and FastQC and MultiQC checked file quality. Trimmomatic was further performed to exclude the low- or poor-quality data according to the parameters (leading, trailing, etc.). Next, HISAT2 software was used to align the sample genome and reference genome. The amount of reading numbers mapped to individual genes were performed by FetureCounts. Differentially expressed genes (DEGs) were normalized by “Trimmed Mean of M-values,” and DESeq2 was normalized by “Relative Log Expression”. A cluster profiler was used to analyze the GO and KEGG pathways from DEGs. Disease Ontology (DO) was generated using the DOSE package, which found the association between DO terms with MeSH, ICD, NCI’s thesaurus, SNOMED, and OMIM. Gene Set Enrichment Analysis (GSEA), and Molecular Signatures Database (MSigDB) were used to identify the enriched gene biological function. According to macrophage polarization gene signatures [33], the M1- and M2-related genes expression was enriched from the DEG data and visualized in heatmaps and volcano plots according to the standard score (Z-score) and log2 ratios between the flavivirus infection group and the mock group. Visualizing data were generated by GraphPad Prism 9.0 (GraphPad Software, San Diego, CA, USA).

### 2.12. Statistical Analysis

Data obtained from three independent experiments, presented as the mean ± standard deviation (SD), were analyzed by unpaired Student’s *t*-test or one-way ANOVA with Tukey’s multiple-comparison test. Statistical significance was set at *p* < 0.05.

## 3. Results

### 3.1. Flavivirus Inoculation Induces Encephalitis-Like Symptoms in a Suckling Mouse Infection Model

**Figure 1:**
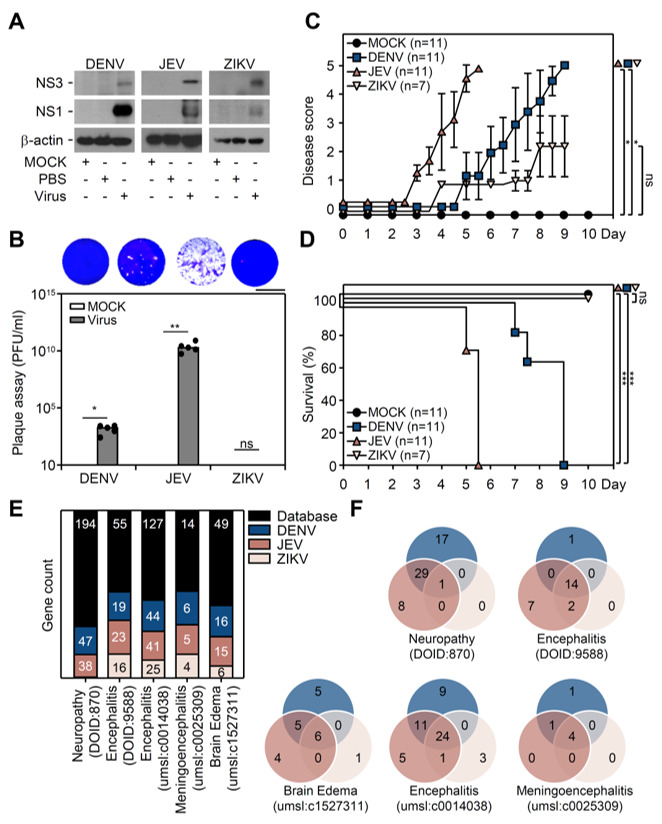
Flavivirus infection induced encephalitis-like symptoms in a suckling mouse model. Seven-day-old neonatal ICR mice were intraperitoneally (i.p.) and intracerebrally (i.c.) injected to inoculate without (*n* = 11, MOCK) or with DENV (*n* = 11, PL046, 1 × 10^6^ pfu), JEV (*n* = 11, RP-9, 1 × 10^4^ pfu), or ZIKV (*n* = 7, PRVABC-59, 1 × 10^6^ pfu). (**A**) Representative blots showed viral nonstructural protein (NS) expression. β-actin was used as the internal control. (**B**) Plaque assay revealed mature virion production at the indicated sacrifice timepoint. (**C**) The disease score showed the progression of disease onset. (**D**) Percentage of survival rate showed mouse mortality after viral infection. (**E**) Disease ontology analysis showed the gene expression profiles related to brain injury in different flavivirus infections. (**F**) Venn diagrams represented the similar expression patterns of a disease-related gene in different flavivirus infections. * *p* < 0.05, ** *p* < 0.01, and *** *p* < 0.001; ns, not significant. Scale bar = 2cm.

To explore the in vivo model of flavivirus-induced encephalitis-like symptoms, our previously exhibited DENV infectious immunocompetent murine model was utilized [32]. Seven-day-old neonatal ICR mice were inoculated with DENV2 PL046 (1 × 10^6^), JEV RP-8 (1 × 10^4^), and ZIKV PRVABC-59 (1 × 10^6^) through intracranial and intraperitoneal injection (Supplementary Figure S1). Infected mice were monitored and sacrificed when they presented paralysis symptoms. To dissect the different infectious efficacy, brain tissues were collected and analyzed by Western blotting. The individual viral nonstructural proteins, NS3 and NS1, expression levels were detected in the flavivirus-infected groups (Figure 1A). The plaque assay further showed that mature virions were significantly (*p* < 0.01) released in brain extracts (Figure 1B). However, no further virions could be detected in the ZIKV group. We also monitored the time-kinetic change in disease score. The criterion of disease score was defined as follows: 0 for health, 1 for reduced mobility, 2 for limbic seizures, 3 for limbic weakness, 4 for paralysis, and 5 for mortality. The quantitative results indicated the progressive disease score (Figure 1C) and the reduced survival rate (Figure 1D) in mice after infection with JEV and DENV, but not ZIKV. A disease ontology analysis was performed to confirm the correlation between patient clinical symptoms and the tested flavivirus infectious models. Disease-related genes were enriched in brain diseases, such as encephalitis, edema, and neuropathy, compared with the established database (accessed on 18 December 2020. http://disease-ontology.org; https://www.disgenet.org/). Quantitative results showed that several disease-related genes, including neuropathy, encephalitis, meningoencephalitis, and brain edema, were upregulated after flavivirus infection (Figure 1E). Venn diagrams showed similar expression patterns, particularly in encephalitis, in three flavivirus infections (Figure 1F). These data indicated that flavivirus JEV and DENV infections in the brain could strongly cause encephalitis-like symptoms.

### 3.2. Flavivirus Infection Induces Neuropathy Accompanied with Neurotoxicity

**Figure 2:**
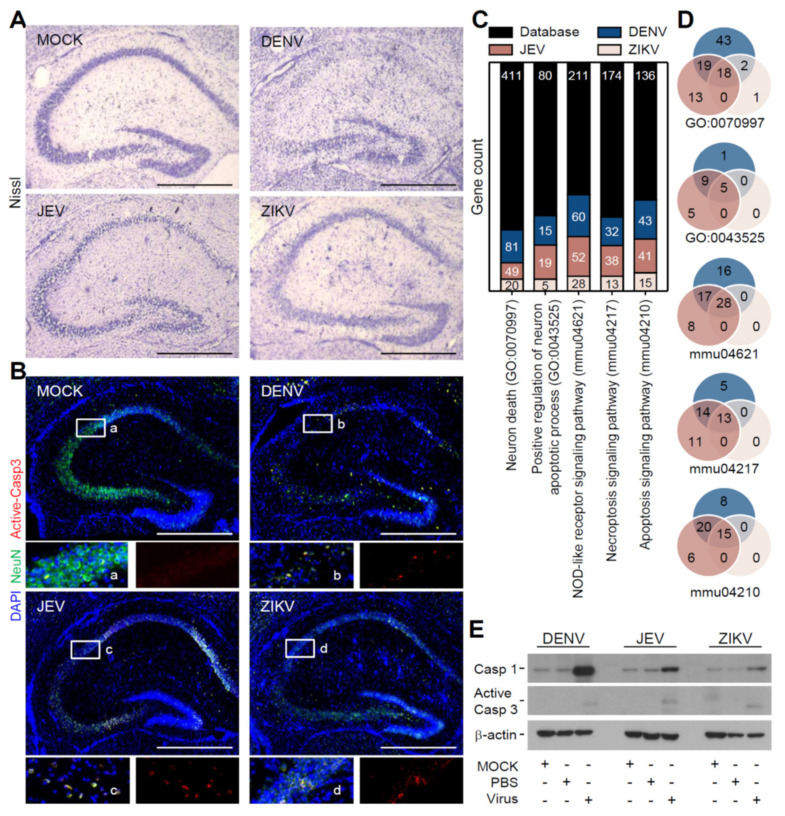
Flavivirus infection caused neuropathy in the hippocampus. Following DENV, JEV, and ZIKV infection in neonatal ICR mice for 5 days, representative images revealed (**A**) Nissl staining and (**B**) fluorescent staining of NeuN (green) plus active caspase (Casp)-3 (red) expression to show neuronal destruction in the hippocampus. DAPI (blue) was stained for nuclei. (**C**) Gene ontology enrichment analysis showed the changed gene expression involving cell death-related pathways. (**D**) Venn diagrams indicated the similarities of death-related gene expression in different flavivirus infections. (**E**) A representative blot showed the expression of caspase (Casp)-1 and active Casp-3. β-actin was used as the internal control. Scale bar = 400 μm.

Our previous works reported neuron destruction caused by DENV infection [17]. To investigate the possible neuropathy and its difference caused by flavivirus infection, histological staining of the Nissl body in the soma of neurons showed decreased neuronal densities in the hippocampus after infection with all three flaviviruses (Figure 2A). Further immunofluorescent staining of active caspase-3, as presented in the NeuN-positive neuronal cells, indicated that cell apoptosis was exhibited in the regions of neuronal destruction after DENV, JEV, and ZIKV infection (Figure 2B). To further elucidate the general cell death caused by flavivirus infection in the brain, transcriptome sequencing (RNA-Seq) analysis was performed and showed the number of cell death-related genes that were enriched in different types of cell death pathways, such as neuronal apoptosis, necroptosis, and pyroptosis (Figure 2C). A similar gene expression pattern indicated that flavivirus infection induced remarkably similar cell death pathways, but to varying degrees (Figure 2D). A NOD-like receptor signaling pathway was the primary responsive target caused by all three flavivirus infections among these upregulated genes. Furthermore, the protein levels also showed that pyroptosis-associated caspase-1 expression and apoptosis-associated caspase-3 activation were significantly induced after infection (Figure 2E). These data show that flavivirus infection causes mouse neuropathy accompanied by the induction of neurotoxicity and multiple cell death pathways.

### 3.3. Flavivirus Infection Causes Peripheral Macrophage Infiltration

**Figure 3:**
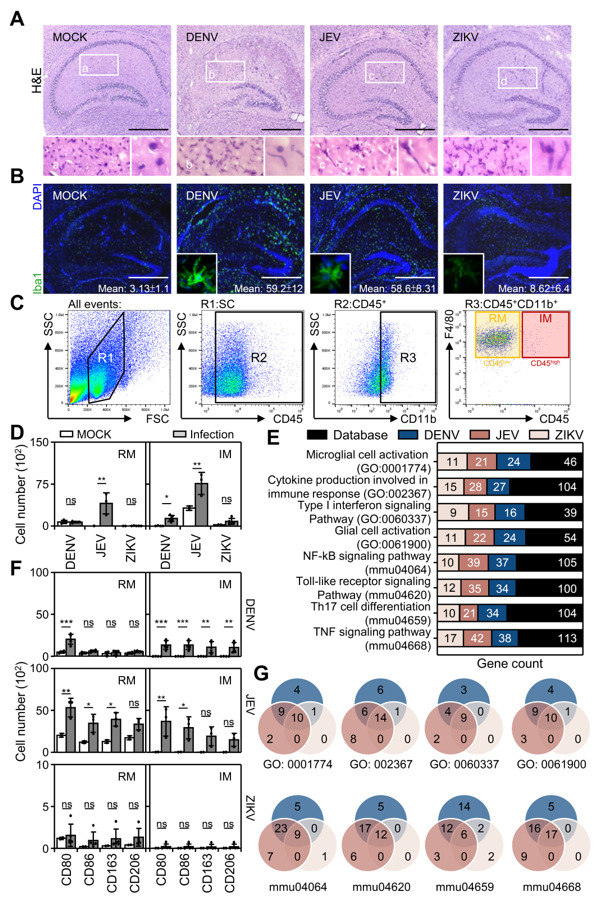
Flavivirus infection resulted in peripheral immune cell infiltration and inflammatory activation in the brain. In DENV-, JEV-, and ZIKV-infected hippocampi, hematoxylin & eosin staining (H&E) showed cell infiltration (**A**). (**B**) Immunofluorescent staining of Iba1 (green) indicated macrophage expression. DAPI (blue) was stained for nuclei. (**C**) The gating strategy in specific regions (R1-3) used to identify resident macrophages or microglia (RM, CD45^low^CD11b^+^) and infiltrated macrophages (IM, CD45^high^CD11b^+^). (**D**) The quantitative number of RMs and IMs in DENV-, JEV-, and ZIKV-infected brains (*n* = 3). (**E**) Immunoprofile of macrophage polarization toward type 1 (CD80 and CD86) or type 2 (CD163 and CD206) in the RM and IM of infected mice (*n* = 3). * *p* < 0.05, ** *p* < 0.01, and *** *p* < 0.001; ns, not significant. (**F**) Gene ontology enrichment analysis showed the changed gene count involving the inflammatory activation pathway. (**G**) Venn diagrams showed the commonality of inflammatory gene expression in three flavivirus infections. Scale bar = 400 μm.

Numerous works in the literature have shown that blood-brain barrier disruption caused peripheral immune cell infiltration during JEV infection [26,29]. Therefore, we next investigated whether peripheral immune cells also participate in DENV-, JEV-, and ZIKV-induced neuropathy in the brain. While Nissl staining showed disrupted neuronal cells in the hippocampus (Figure 2A), H&E staining confirmed that histology and cell morphology were significantly changed after flavivirus virus infection. More importantly, in infected cases, the cell nucleus was present in an irregular elongated shape compared with the MOCK group (Figure 3A), and it was highly possible that the elongated cells were macrophages, known as microglia. To verify the H&E results, immunofluorescent staining of Iba1, a common protein specifically expressed in microglia/macrophages, indicated that flavivirus infection caused an increase in microglia/macrophages (Figure 3B).

To further investigate the immune cell phenotype in the infected brain, multicolor immunostaining followed by flow cytometric analysis was used to dissect the immune cell profile in CD45-positive cells isolated from the mouse brains. A gating strategy showed that CD45^+^CD11b^+^ cells were determined to be the monocyte population. The F4/80 and CD45 expression levels further distinguished resident microglia (RM, CD45^low^CD11b^+^) and infiltrated macrophages (IM, CD45^high^CD11b^+^) (Figure 3C). The qualitative results revealed that the RM cell number significantly increased only in the JEV infection group. However, the IM cell number significantly increased in the DENV and JEV infection groups (Figure 3D). In the ZIKV-infected group, no further changes in RM and IM were observed. Notably, compared with M2-related markers (CD163 and CD206), M1-related markers (CD80 and CD86) were significantly increased in RM and IM cells after DENV and JEV infection, while only DENV infection also upregulated a partial increase in M2 (Figure 3E). Further RNA-Seq analysis confirmed the findings that both DENV and JEV caused an increase in M1-related inflammatory gene activation (Figure 3F) and that a similar gene pattern was commonly induced by DENV and JEV (Figure 3G). These results indicated that flavivirus infection increased expression and M1 polarization in resident microglia, and infiltrated macrophages to facilitate M1-related immune responses.

### 3.4. Flavivirus Infection Facilities Type-1 Dominated Immune Activation

**Figure 4:**
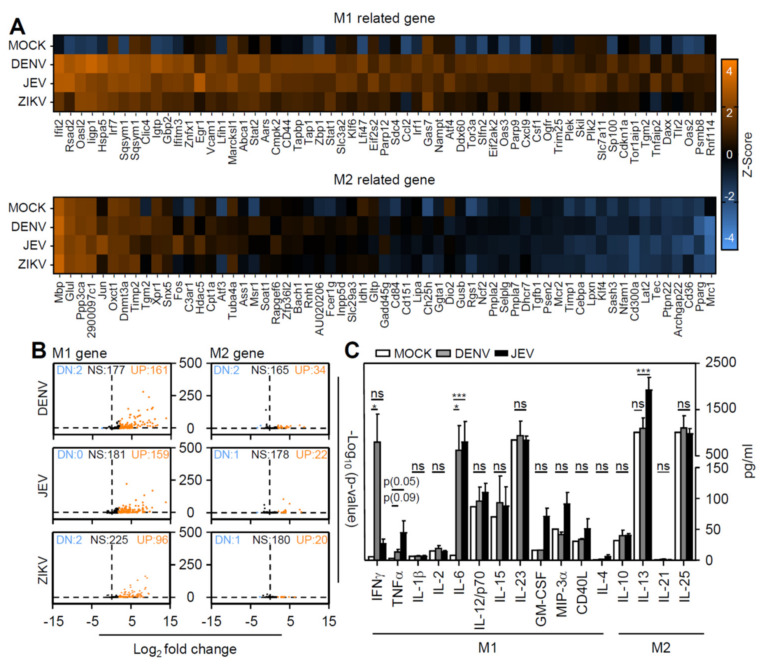
Flavivirus infection induced type 1 macrophage polarization and immune responses in the brain. In DENV-, JEV-, and ZIKV-infected brains, (**A**) representative heat map of the top 61 M1-related and M2-related gene expression. (**B**) The volcano plot showed the number of upregulated (UP), nonsignificant (NS), and downregulated (DN) genes after viral inoculation. (**C**) Multiplex assay measured M1- and M2-related cytokine/chemokine production (pg/mL) in DENV- and JEV-infected brains. * *p* < 0.05 and *** *p* < 0.001; ns, not significant.

According to immune profiling and inflammatory gene expression profiles, DENV and JEV infection caused macrophage polarization toward the M1 phenotype (Figure 3). To further validate the immune phenotype of inflamed brains infected with a flavivirus, whole-brain extracts were collected, and RNA-seq was performed. Visualizing heatmap results revealed increased gene expression in the M1-related gene set, but not the M2-related gene set (Figure 4A). Volcano plot results further confirmed the increased M1-related gene expression after infection (Figure 4B). However, the significant induction of the M1-related immune response consisted of DENV and JEV infection, but not ZIKV infection. We next performed a multiplex assay to detect the production of cytokines and chemokines in the brains of DENV- and JEV-infected mice. Compared with M2-related cytokines, M1-related cytokines, including IFN-γ, TNF-α, and IL-6, were significantly increased after DENV and JEV infection (Figure 4C). These results indicated that flavivirus infection induced M1 polarization accompanied by an M1-dominant inflammatory response.

### 3.5. Inhibition of Peripheral Immune Cell Invasion and TNF-α Reduces Resident M1 Polarization

**Figure 5:**
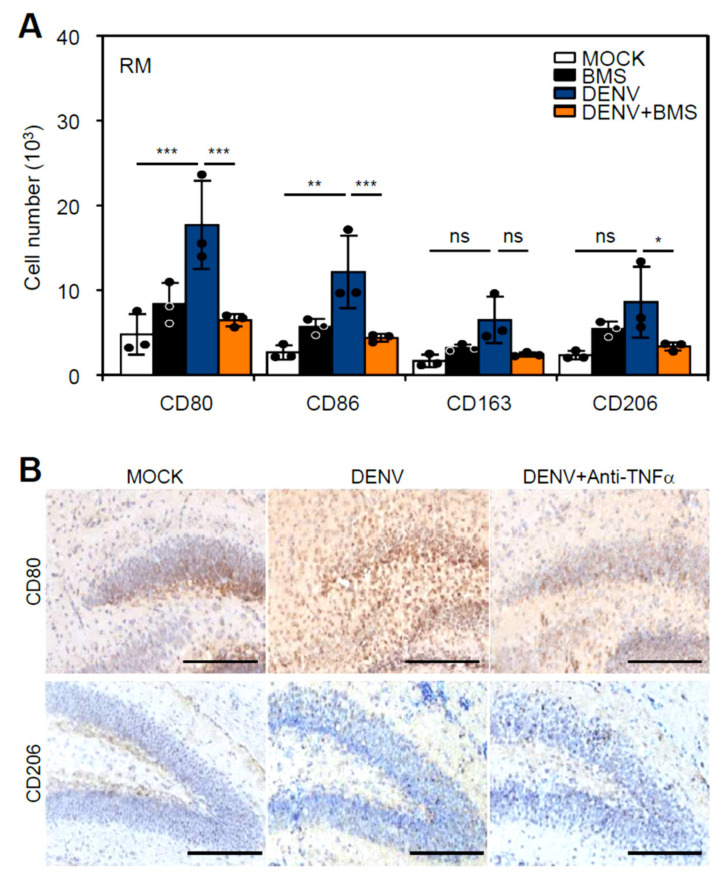
Pharmacologically inhibiting CCR2 and TNF-α reduced resident microglial polarization. Neonatal ICR mice were kinetically pretreated with the CCR2 antagonist BMS (10 mg/kg) for one day, followed by further treatment at day 2 and 5, accompanied by inoculation with or without DENV at day 0. (**A**) Immunoprofile of macrophage polarization toward type 1 (CD80 and CD86) or type 2 (CD163 and CD206) in the RM of DENV-infected mice (*n* = 3). * *p* < 0.05, ** *p* < 0.01, and *** *p* < 0.001; ns, not significant. (**B**) Furthermore, immunohistochemistry staining of CD80 or CD206 (brown) showed the expression of M1- or M2-related macrophages in DENV-infected mice with or without anti-TNF-α (250 μg/kg) treatment. Scale bar = 200 μm.

While infiltrated macrophages may also cooperate with microglia to expand CNS inflammation, we next evaluated the therapeutic effects by retarding CCR2-mediated peripheral macrophage infiltration [34,35]. Seven-day-old neonatal ICR mice were pretreated with the CCR2 antagonist BMS (10 mg/kg) before infection with DENV2 PL046 (1 × 10^6^) and then received an extra dosage of BMS at 2 days post-infection (dpi) and 5 dpi (Supplementary Figure S2A). The quantitative results showed no significant decrease in disease progression (Supplementary Figure S2B) but a slight reversed effect on the survival rate (Supplementary Figure S2C) in mice after DENV infection with BMS treatment. Flow cytometry-based immune profiling confirmed that the blockade of CCR2 inhibited M1 polarization (Figure 5A). Based on the requirement of TNF-α in mediating M1 polarization [36] and our previous work, anti-TNF-α treatment significantly reduced DENV-induced neurotoxicity [17]. In DENV-infected mice treated with anti-TNF-α, immunohistochemical staining showed that anti-TNF-α treatment retarded M1 polarization, as shown by the measurement of CD80- and CD206-positive cells (Figure 5B). These results indicated that pharmacologically inhibiting CCR2 and TNF-α effectively inhibited DENV-induced M1 polarization.

## 4. Discussion

**Figure 6:**
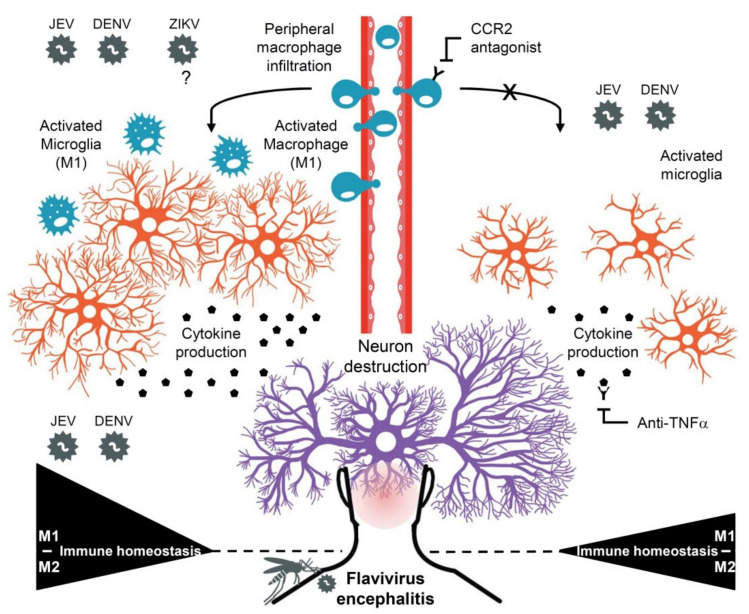
A hypothetical model of type 1 macrophage polarization and immune responses involved in JEV- and DENV-induced encephalitis. Flavivirus infections, such as JEV and DENV, but not ZIKV, in the brain could cause macrophage infiltration into the CNS, followed by type 1 polarization under the encephalitic microenvironment. In cooperation with polarized resident microglia, type 1 macrophages and their immune responses may have exacerbated neurotoxicity and neuropathic severity, while blockading CCR2, which is critical for peripheral macrophage infiltration, and TNF-α may confer neuroprotection against M1 polarization.

This study first revealed that DENV infection could induce viral encephalitis by causing macrophage infiltration in the brain, the development of M1 macrophage polarization, and its related inflammatory responses. This phenomenon was similar to JEV, a well-known flavivirus that quickly caused viral encephalitis [37]. What is puzzling is that ZIKV did not cause virus replication in the brain or induce severe encephalitis-like symptoms compared with JEV or DENV. It needs further validation regarding the model used in this study. These findings showed that flavivirus infections, such as JEV and DENV, in the brain can cause various encephalitis-like symptoms, probably by causing brain infiltration of peripheral blood macrophages followed by the induction of macrophage polarization in infiltrated macrophages and resident microglia in the brain. However, based on the current findings, it was difficult to conclude that ZIKV caused minor effects on viral encephalitis regarding its different effects on microglial activation toward M1 polarization. This research also revealed that blocking treatment with targeted CCR2 and TNF-α could partially alleviate the polarization of M1 macrophages. All results and possible pathological significance and treatment strategies were summarized in Figure 6.

As clinical and biomedical research reported, JEV, a neurotropic flavivirus, can infect and replicate in neuronal cells [38]. This study proved that JEV infection caused severe encephalitic symptoms and high mortality in mice. While ZIKV also infected the brain and disrupted neuronal development due to its pathogenicity [39], there was no effect in this study’s mouse infection model. It is speculated to be related to the difference in the infectious models and the selectivity of the virus strain, and further experiments should be required to distinguish or verify it accordingly. In the study of DENV infection, as our previous results showed, DENV could also cause brain infection and virus replication followed by the development of encephalitis-like symptoms after infection [12,32]. Furthermore, the results of RNA-Seq studies have shown that all three flaviviruses can induce encephalitis-associated gene upregulation, indicating the pathology of flavivirus encephalitis. Based on the above results, the difference between ZIKV and DENV in brain infections, especially in neurotropic activity, needs further investigation, although neurotoxicity-associated caspase-3 and neuroinflammation-associated caspase-1 are all caused by these two viruses. In addition, whether the three flaviviruses have different infectivities and effects on non-neuronal cells also needs to be explored.

Generally, brain viral infections often cause neurotoxicity or indirectly lead to neuronal cell damage through inflammatory responses driven by non-neuronal cells in the brain [17,40]. The cytopathological results of the three flaviviruses in this study showed that neuronal cells were lost or damaged in the hippocampus. Cell apoptosis may occur in damaged hippocampal neuronal cells and lead to neuropathological damage in mice. Related gene expression and bioinformatics analysis showed several cell death signaling-pathways, including apoptosis, cell necrosis, and inflammation-related pyroptosis. Past studies on JEV and dengue infection have confirmed that the above cell death was related to the pathogenicity of the virus infection [41,42,43,44,45,46,47,48]. In addition, flavivirus infection led to cell pyroptosis and often expanded proinflammatory responses to deteriorating tissue/organ function [49,50]. This study also confirmed that pyroptosis occurred in mouse brain infections, while it increased in proinflammatory genes and caspase-1 indicated the activation of the inflammasome during pyroptosis, which was closely related to encephalitis. These results showed that all flaviviruses caused similar post-infection effects in the brain, including neurotoxicity accompanied by the progression of CNS inflammation and encephalitis.

Neuroinflammation is the immunopathological basis of viral encephalitis [26,51]. According to the results of this study, flavivirus infection of the brain increased the expression and activation of immune cells in the brain. Immunostaining of brain tissue sections can verify specific activated immune cell biomarkers, such as Iba-1 [52]. This study further used flow cytometry to monitor the types of immune cell populations infiltrating the brains of mice. In addition to the original resident microglia, infiltration of peripheral blood macrophages was also increased. The amount of macrophage infiltration was related to encephalitic pathology and fatality rate, mainly caused by JEV and DENV. Notably, JEV and DENV brain infections polarized M1 microglia/macrophages. M1 microglia/macrophage polarization in the brain was significantly related to neuronal degeneration-related diseases [49,50,53,54]. Therefore, the results of this study linked M1 microglia/macrophage polarization induced by flavivirus infection and worsened the occurrence of encephalitis. However, polarization based on M1 macrophages was related to cellular inflammation and biological functions, such as immune defense, which included antimicrobial and anticancer functions [55,56,57,58]. Our laboratory’s previous results showed that the depletion of microglial cells in the brain would worsen DENV infection in the brains of mice, leading to a higher incidence of encephalitis and mortality rate [12]. Accordingly, microglial cells in the brain may play an APC-like role in antiviral defenses. The polarization of M1 microglia/macrophages should be further explored to determine the physiological and pathological effects of flavivirus infection in the brain.

This study also provided more evidence to show that flavivirus infection could induce the polarization of M1 microglia/macrophages in the brain. The implementation and analysis of RNA-Seq indicated that the gene expression pattern of type 1 immunity supported the results of immunostaining and immune monitoring in brain tissue. The polarization of M1 microglia/macrophages could change the performance of immune factors in the microenvironment and induce the polarization of type 1 immunity to be linked to the occurrence of neurodegenerative diseases [49,50,53,54]. Based on our findings, immune factors such as IFN-γ/TNF-α/IL-6 could be induced by JEV and DENV infection. We speculated that these immune factors could all induce proinflammatory-related type 1 immunity. In turn, it caused irreversible encephalitis symptoms and neurotoxicity in the mouse brains. In contrast, targeting type 1 immunity-related factors should be regarded as a possible therapeutic strategy to ameliorate the occurrence of encephalitis. Indeed, studies on anti-encephalitis caused by JEV infection showed that targeting CCR2 and other inflammatory cytokines/chemokines could all improve the occurrence of encephalitic disease progression [40,59]. Based on this hypothesis, we have previously verified that the target TNF-α could reduce the occurrence of DENV-induced encephalitis-like symptoms in mice [17]. However, the roles of CCR2 and TNF-α should be validated by using more doses and the modified treatment schedule, while this study showed that the blockade of both CCR2 and TNF-α could partially inhibit the polarization of M1 macrophages, which therefore may reduce encephalitis incidences.

Based on the current experimental designs, this study needs to be aware of some limitations and be verified for future research. First, the choice of research models needs to consider the difference in the route of infection, the pathogenicity of the infected virus strains, the susceptibility of the infected hosts, and the neuropathology of the infected brain. Second, the physiopathological significance of the polarization of M1 microglia/macrophages needs to be verified, and more methodologies and research strategies need to be examined for its applicability. Third, in addition to JEV, supporting evidence for the pathogenicity of DENV and ZIKV in clinical encephalitis needs to be confirmed. In conclusion, this study confirmed experimental disease models of viral encephalitis infected with three flaviviruses in the brains of mice. Accompanied by neurotoxicity and neuroinflammation, JEV and DENV can induce microglia and infiltrated macrophages to undergo M1 polarization and related proinflammatory activation. Thus, targeting the occurrence of type 1 immunity may alleviate the pathologically lethal effect of viral encephalitis.

## Data Availability

The data presented in this manuscript are available from the corresponding author upon reasonable request.

## Supplementary Materials

The following are available online at https://www.mdpi.com/article/10.3390/cells10113181/s1, Figure S1: Flavivirus infection induces encephalitis-like symptoms in a suckling mouse model, Figure S2: Pharmacologically inhibiting CCR2 and TNF-α reduces M1 polarization and slightly prolongs mice survival.
