# Supplementary material for: Polarization of Type 1 Macrophages Is Associated with the Severity of Viral Encephalitis Caused by Japanese Encephalitis Virus and Dengue Virus
Source: Cells. 2021 Nov 15;10(11):3181. doi: 10.3390/cells10113181 (PMC8621422; doi:10.3390/cells10113181)

**Supplemental Data**

# Polarization of Type 1 Macrophages is Associated with the Severity of Viral Encephalitis Caused by Japanese Encephalitis Virus and Dengue Virus

Ming-Kai Jhan [1,2], Chia-Ling Chen [3], Ting-Jing Shen [1,2], Po-Chun Tseng [2,4], Yung-Ting Wang [2], Rahmat Dani Satria [2,5,6,7] Chia-Yi Yu [8] and Chiou-Feng Lin [1,2,4,5,*]

**Supplemental Figure 1** Flavivirus infection induces encephalitis-like symptoms in a suckling mouse model. Flowchart of the experimental design showed seven-day-old neonatal ICR mice intraperitoneally (*i.p.*) and intracerebrally (*i.c.*) injected to inoculate without (n=11, MOCK) or with DENV (n=11, PL046, $1\times10^6$ pfu), JEV (n=11, RP-9, $1\times10^4$ pfu), or ZIKV (n=7, PRVABC-59, $1\times10^6$ pfu).

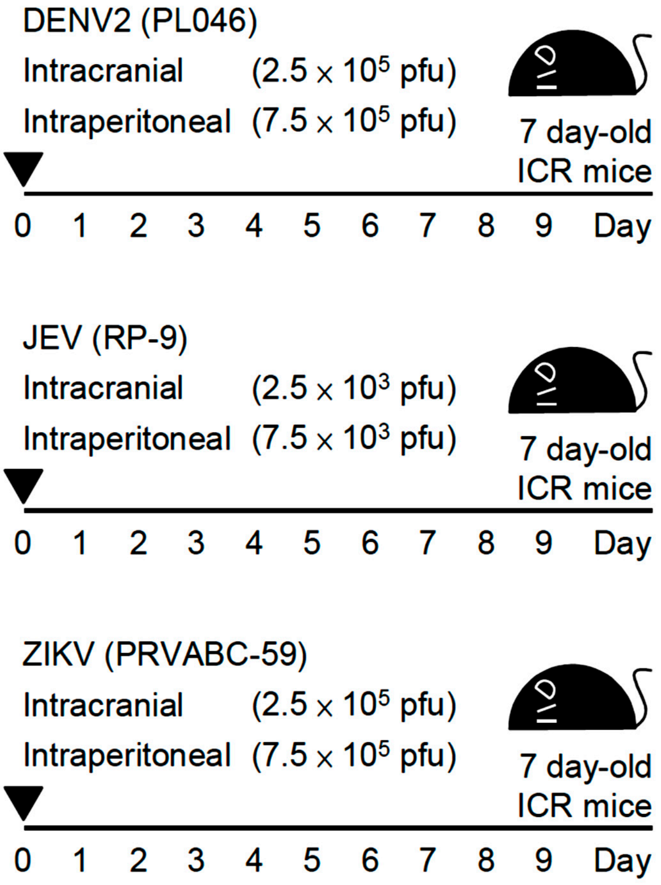

**Supplemental Figure 2** Pharmacologically inhibiting CCR2 and TNF-α reduces M1 polarization and slightly prolongs mice survival. **(A)** Flowchart of the experimental design showed neonatal ICR mice kinetically pretreated with the CCR2 antagonist BMS (10 mg/kg) for one day followed by further treatment at days 2 and 5 accompanied by inoculation with or without DENV at day 0. **(B)** The disease score indicates the progression of disease onset. **(C)** Percentage of survival rate showing mouse mortality following viral inoculation. * *P* <0.05, ** *P* <0.01, and *** *P* <0.001. ns, not significant.

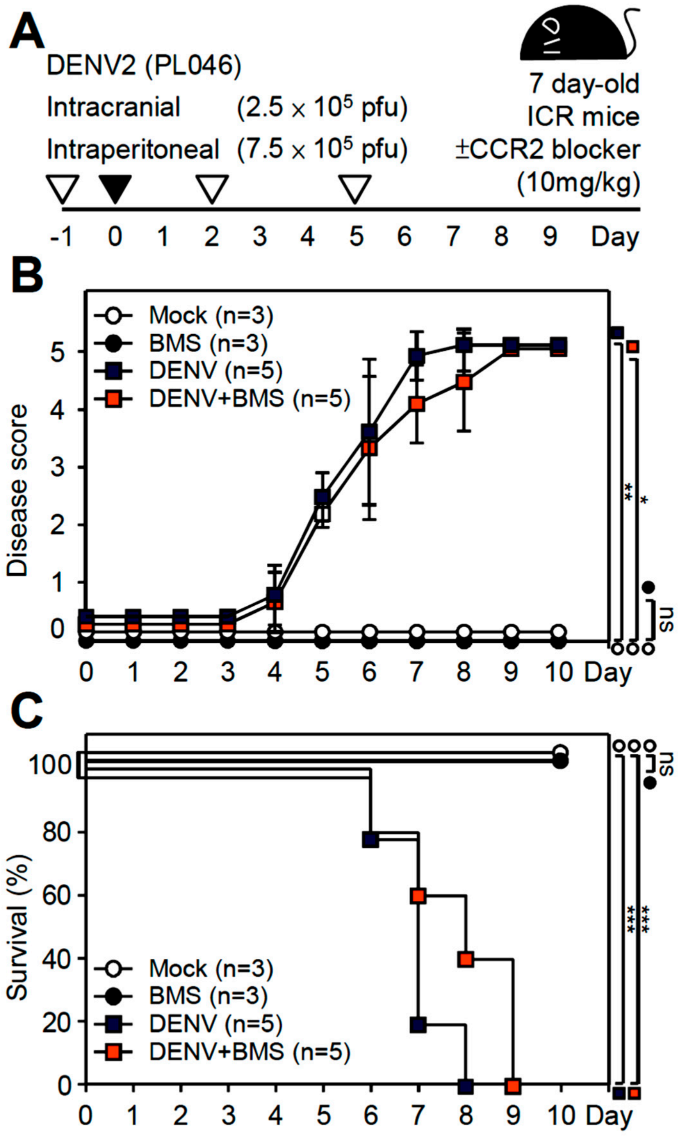

Supplement: Supplementary file 1 [file cells-10-03181-s001.zip › cells-1432574-supplementary.pdf]
